# Two Novel Phenolic Compounds from the Rhizomes of *Cyperus rotundus* L*.*

**DOI:** 10.3390/molecules171112636

**Published:** 2012-10-25

**Authors:** Zhongliu Zhou, Wenqing Yin

**Affiliations:** 1 Chemistry Science and Technology School, Zhanjiang Normal University, 29 Cunjin Road, Zhanjiang 524048, China; 2 School of Chemistry & Chemical Engineering of Guangxi Normal University, Ministry of Education Key Laboratory of Chemistry and Molecular Engineering of Medicinal Resource, Guilin 541004, China; Email: Yinwq0000@163.com

**Keywords:** *Cyperus rotundus* L., phenolic compounds, isolation, characterization

## Abstract

Two novel compounds, 1*α*-methoxy-3*β*-hydroxy-4*α*-(3′,4′-dihydroxyphenyl)-1, 2,3,4-tetrahydronaphthalin (**1**) and 1*α*,3*β*-dihydroxy-4*α*-(3′,4′-dihydroxyphenyl)-1,2,3,4-tetrahydronaphthalin (**2**), were isolated along with six known compounds **3**–**8** from the rhizomes of *Cyperus rotundus*. This paper reports the isolation and full spectroscopic characterization of these new compounds by NMR, UV, IR and MS data.

## 1. Introduction

*Cyperus rotundus* L. is a weed which is well distributed in the temperate tropical and subtropical regions of the World. The tuber of *Cyperus rotundus* is a kind of Traditional Chinese Medicine named “Xiangfuzi”, which is widely used in folk medicine as an antidiarrheal, antidepressant, anti-*Candida*, antipyretic, analgesic, anti-inflammatory, and anti-emetic remedy for dysentery and women’s diseases [1,2]. Previous phytochemical studies on this plant have revealed the presence of alkaloids, flavonoids, glycosides and furochromones, and many new sesquiterpenoids [2,3,4,5,6]. Recently, we reported a new flavonoid and five known compounds from the rhizomes of *Cyperus rotundus* [7]. In continuation of our search for new biologically active compounds from Chinese medicinal plants, we have further phytochemically investigated the rhizomes of this plant, resulting in the isolation two new components, 1*α*-methoxy-3*β*-hydroxy-4*α*-(3′,4′-dihydroxyphenyl)-1,2,3,4-tetrahydronaphthalin (**1**) and 1*α*,3*β*-di-hydroxy-4*α*-(3′,4′-dihydroxyphenyl)-1,2,3,4-tetrahydronaphthalin (**2**), together with six known compounds **3**–**8**. The present paper deals with the experimental details of separation and structure elucidation of the constituents of the compounds **1**–**8**.

## 2. Results and Discussion

**Figure 1:**
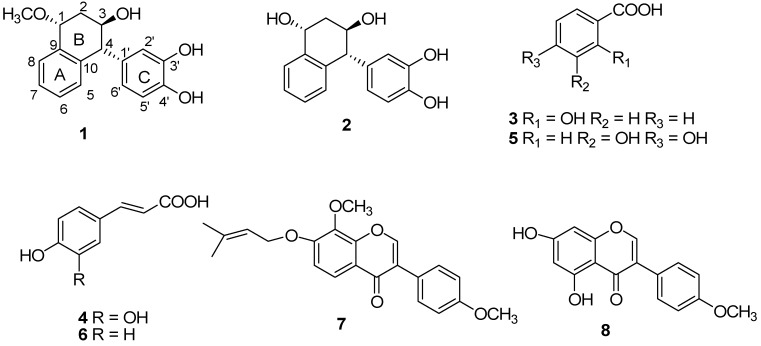
Chemical structures of compounds **1**–**8** isolated from the rhizomes of *Cyperus rotundus* L.

The phytochemical study of 95% ethanol extract obtained from the rhizomes of *Cyperus rotundus* L. afforded eight compounds, including two new constituents and six known compounds (Figure 1). The structures of compounds **1**–**8** were elucidated by detailed spectroscopic analysis and comparison of their spectroscopic data with those reported in the literature.

**Figure 2:**
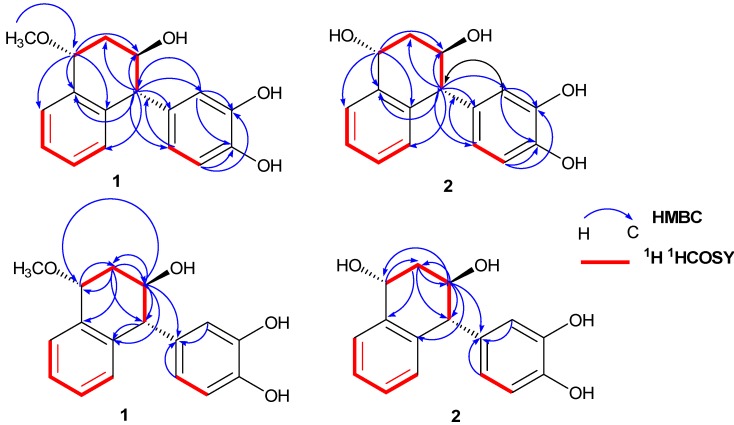
Key HMBC and ^1^H ^1^H-COSYcorrelations of **1** and **2**.

Compound **1**, had the molecular formula C_17_H_18_O_4_, as deduced from the positive-ion HR-ESI-MS (*m*/*z* 309.1107 [M+Na]^+^) and ^13^C-NMR spectrum. The IR spectrum displayed absorptions at 3200–3450, 1603, and 1521 cm^−1^, consistent with the presence of hydroxyl and phenyl groups, respectively. The occurrence of a 1,2-disubstituted phenyl (A ring) in the molecule could be easily deduced from the ^1^H- and ^13^C-NMR spectra [δ 7.04 (1H, d, *J* = 7.8 Hz, H-5), 7.14 (1H, t, *J* = 7.8 Hz, H-6), 7.15 (1H, t, *J* = 7.8 Hz, H-7), and 7.43 (1H, d, *J* = 7.8 Hz, H-8); δ 139.1 (C-9), 137.9 (C-10), 128.9 (C-5), 127.6 (C-6), 126.9 (C-8) and 125.4 (C-7)] (Table 1 and Table 2). The detailed 2D NMR analysis of ^1^H^1^H COSY, HMQC, and HMBC correlations also implied that **1** had a 1,2-disubstituted phenyl (A ring) (Figure 2). Taking into account the nine degrees of unsaturation, **1** must include a six-membered ring (B ring). This was revealed by the HH correlations of the spin system H-1/H_2_-2/H-3/H-4 as well as the HMBC correlations from H-1 to C-2, C-3, C-8, C-9, and C-10, from H-4 to C-3, C-9, C-10, and C-2, from H-3 to C-2, C-4, C-10, and C-1, and from H-2 to C-1, C-3, C-9, and C-4 (Figure 2). Thus, the skeleton of **1** was believed to be a 1,2,3,4-tetrahydronaphthalin (ring A and ring B). 

**Table 1:** ^1^H-NMR (400 MHz, in CD_3_OD) spectroscopic data of **1**–**2**.

Position	1	2
1	4.91 (1H, m)	5.03 (1H, m)
2	1.91–2.03 (2H, m)	1.87–1.93 (2H, m)
3	3.79 (1H, m)	3.83 (1H, m)
4	4.09 (1H, d, *J* = 6.8 Hz)	4.11 (1H, d, *J* = 6.7 Hz)
5	7.04 (1H, d, *J* = 7.8 Hz)	7.06 (1H, d, *J* = 7.6 Hz)
6	7.14 (1H, t, *J* = 7.8 Hz)	7.16 (1H, t, *J* = 7.6 Hz)
7	7.15 (1H, t, *J* = 7.8 Hz)	7.16 (1H, t, *J* = 7.6 Hz)
8	7.43 (1H, d, *J* = 7.8 Hz)	7.39 (1H, d, *J* = 7.6 Hz)
2′	6.73 (1H, d, *J* = 2.0 Hz)	6.75 (1H, d, *J* = 2.0 Hz)
5′	6.79 (1H, d, *J* = 8.0 Hz)	6.77 (1H, d, *J* = 8.0 Hz)
6′	6.68 (1H, dd, *J* = 8.0, 2.0 Hz)	6.65 (1H, dd, *J* = 8.0, 2.0 Hz)
OCH_3_	3.31 (3H, s)	-

**Table 2:** ^13^C-NMR (100 MHz, in CD_3_OD) spectroscopic data of **1**–**2**.

Position	1	2	Position	1	2
1	74.1	65.8	10	137.9	138.1
2	38.1	37.9	1′	134.8	134.8
3	66.3	66.1	2′	116.3	116.3
4	49.8	49.7	3′	145.6	145.7
5	128.9	128.9	4′	143.1	143.1
6	127.6	127.4	5′	116.0	115.8
7	125.4	125.3	6′	122.4	122.6
8	126.9	126.5	OMe	57.3	-
9	139.1	139.4			

**Figure 3:**
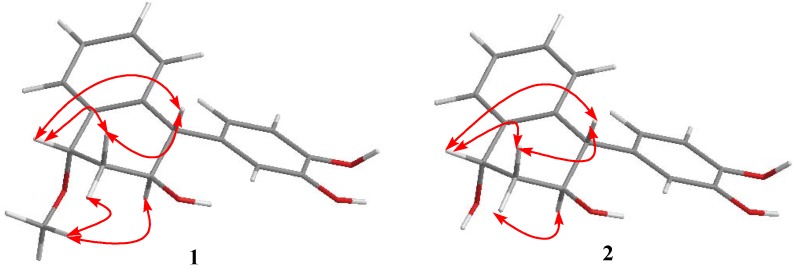
Selected ROESY correlations of **1** and **2**.

The ^1^H and ^13^C-NMR spectrum of **1** showed the presence of an aromatic ABX system [δ 6.73 (1H, d, *J* = 2.0 Hz, H-2′), 6.79 (1H, d, *J* = 8.0 Hz, H-5′), and 6.68 (1H, dd, *J* = 2.0, 8.0 Hz, H-6′); δ (C) 145.6 (C-3′), 143.1 (C-4′), 134.8 (C-1′), 122.4 (C-6′), 116.3 (C-2′), and 116.0 (C-5′)] (Table 1 and Table 2), suggesting the presence of a 1′,3′,4′-trisubstituted phenyl in the molecule. Considering the molecular formula of **1**, two hydroxyl groups should be attached to C-3′ and C-4′, respectively. Thus, compound **1** contained a 3′,4′-dihydroxyphenyl group (ring C). Moreover, the 3′,4′-dihydroxyphenyl group (ring C) was attached to the C-4 position of the ring B, which was supported by the HMBC correlations between H-3 (δ 3.79) and C-4 and C-1′, H-4 (δ 4.09) and C-1′, C-2′, and C-6′, H-2′ (δ 6.73) and C-1′ and C-4, and H-6′ (δ 6.68) and C-1′ and C-4 (Figure 2). In the B ring, a methoxyl group was located at C-1 based on the HMBC correlations from the methoxyl protons (δ 3.31) to C-1 (δ 74.1) and a hydroxyl group was found at C-3 based on the HMBC correlations of H-3 (δ 3.79) to C-1, C-4, and C-10. The *J* value between the protons of H-3 and H-4 (*J* = 6.8 Hz) was consistent with the stereochemistry of H-3*α* and H-4*β*. The absence of a ROESY correlation between H-3 and H-4 also suggested a nearly antiperiplanar arrangement of these two protons. The ROESY correlation of H-1 to H-4 established the stereochemistry of H-1*β* and H-4*β* (Figure 3). Therefore, **1** was assigned as 1*α*-methoxy-3*β*-hydroxy-4*α*-(3′,4′-dihydroxyphenyl)-1,2,3,4-tetrahydronaphthalin, and named methoxycyperotundol.

The molecular formula of compound **2** was determined to be C_16_H_16_O_4_ by the positive ion at *m/z* 295.0948 [M+Na]^+^ in the HRESIMS. Its IR spectrum displayed absorptions attributable to hydroxyl (3300-3500 cm^−1^) and phenyl groups (1607, 1518 cm^−1^). The ^1^H and ^13^C-NMR spectroscopic data of **2** were similar to those of **1**, with the exception of a methoxyl group (δ 3.31; δ 57.3) at C-1 in **1**, instead of a hydroxyl in **2** (Table 1 and Table 2). The suggestion was in accord with the observation of the upfield shift of C-1 signal from δ 74.1 in **1** to δ 65.8 in **2** (Table 2). This was further established by the HMBC correlations from H-1 to C-8, C-10, C-2, and C-3 (Figure 2). Therefore, compound **2** was identified as 1*α*,3*β*-dihydroxy-4*α*-(3′,4′-dihydroxyphenyl)-1,2,3,4-tetrahydronaphthalin, and named cyperotundol.

The structures of the other isolated components: salicylic acid (**3**), caffeic acid (**4**), protocatechuic acid (**5**), *p*-coumaric acid (**6**), pongamone A (**7**) and biochanin A (**8**) were determined by comparison with the ^1^H- and ^13^C-NMR spectral data in the literature [8,9,10,11]. To the best of our knowledge, the known compounds, pongamone A (**7**) and biochanin A (**8**) in the rhizomes of *Cyperus rotundus* are reported for the first time.

## 3. Experimental

### 3.1. General

UV spectra were recorded on a Hewlett-Packard HP-845 UV-VIS spectrophotometer. IR spectra were recorded on a Nicolet 470 spectrometer and MS on a Varian MAT-212 mass spectrometer and a Shimadzu GC-MS model QP2010 Plus spectrophotometer, respectively. NMR spectra were recorded on a Bruker AM-400 spectrameter (400 MHz for ^1^H-NMR, 100 MHz for ^13^C-NMR) using standard Bruker pulse programs. Chemical shifts are given as δ values with reference to tetramethylsilane (TMS) as internal standard. Column chromatography separations were carried out on silica gel (200–300 mesh, Qingdao Haiyang Chemical Co. Ltd, Qingdao, China), ODS (50 mesh, AA12S50, YMC), Diaion HP-20 (Pharmacia, Peapack, NJ, USA) and Sephadex LH-20 (Pharmacia, Peapack, NJ, USA). All other chemicals used were of biochemical reagent grade.

### 3.2. Plant Material

The rhizomes of *Cyperus rotundus* were collected in Zhanjiang, Guangdong Province of China in September 2009, and were identified by one of the authors (Wen-qing Yin of the School of Chemistry & Chemical Engineering of Guangxi Normal University, Ministry of Education Key Laboratory of Chemistry and Molecular Engineering of Medicinal Resource, Guilin, China). A voucher specimen (No.20090903) has been deposited in the authors’ laboratory.

### 3.3. Extraction and Isolation

The dry rhizomes of *Cyperus rotundus* (10 kg) were extracted three times under reflux with 95% EtOH (150 L × 2 h). After removing the solvent under reduced pressure, the residue was suspended in water and then sequentially extracted with petroleum ether, CH_2_Cl_2_, EtOAc and *n*-BuOH. The EtOAc extract (88 g) was subjected to silica gel column chromatography (CC) using CHCl_3_-MeOH mixtures (1:0 to 0:1) and divided into eight main fractions by TLC detection. Fraction 5 was separated by CC over silica gel using CHCl_3_-MeOH (6:1) and Sephadex LH-20 CC using CHCl_3_-MeOH (1:1) to afford **7** (19 mg) and **8** (23 mg). Fraction 7 was chromatographed on silica gel eluting with CHCl_3_-MeOH-H_2_O (9:1:0.1 to 7:3:0.5) and ODS silica gel with MeOH-H_2_O (1:1 to 1:0) to furnish **1** (13 mg) and **2** (18 mg). Fraction 8 was separated by CC on Si gel using CHCl_3_-MeOH (10:1) to give subfraction 8-1 (6.1 g), subfraction 8-2 (10 g) and subfraction 8-3 (4 g). Subfraction 8-2 was purified by semi-preparative HPLC to afford compounds **3** (7 mg), **4** (9 mg), **5** (9 mg), and **6** (11 mg). 

### 3.4. Characterization of Methoxycyperotundol (**1**)

Obtained as colorless needles, [α]^25^_*D*_: −51.9° (*c* 0.5, MeOH); UV λ_max_ (MeOH): 276 nm; IR *v*_max_ (KBr): 3200–3450 cm^−1^, 1603 cm^−1^ and 1521 cm^−1^. HR-ESI-MS *m*/*z* 309.1107 (C_17_H_18_O_4_Na [M+Na]^+^, Cal. 309.1103). ^1^H-NMR and ^13^C-NMR (CD_3_OD) data see Table 1 and Table 2.

### 3.5. Characterization of Cyperotundol (**2**)

Obtained as colorless needles. [α]^25^_*D*_: −89.4° (*c* 0.1, MeOH). UV (MeOH) λ_max_: 279 nm. IR *v*_max_ (KBr): 3300–3500 cm^−1^, 1607 cm^−1^, and 1518 cm^−1^. HR-ESI-MS *m*/*z* 295.0948 ([M+Na]^+^, Calcd. for C_16_H_16_O_4_, 295.0946). ^1^H-NMR and ^13^C-NMR (CD_3_OD) data see Table 1 and Table 2.

## 4. Conclusions

During the phytochemical survey of the rhizomes of *Cyperus rotundus*, two novel constituents methoxycyperotundol (**1**) and cyperotundol (**2**) were obtained, along with six known components. Additionally, the known compounds, pongamone A (**7**) and biochanin A (**8**) in the rhizomes of *Cyperus rotundus* L are reported for the first time.
